# Insects as alternative feed for ruminants: comparison of protein evaluation methods

**DOI:** 10.1186/s40104-021-00671-2

**Published:** 2022-02-11

**Authors:** Pablo G. Toral, Gonzalo Hervás, Mariana Gabriela González-Rosales, Alejandro G. Mendoza, Lizbeth E. Robles-Jiménez, Pilar Frutos

**Affiliations:** 1 Instituto de Ganadería de Montaña (CSIC-University of León), Finca Marzanas s/n, 24346 Grulleros, León, Spain; 2 Facultad de Medicina Veterinaria y Zootecnia, Universidad Autónoma del Estado de México, No. 100 Instituto Literario 100, Col. Centro, 50000 Toluca, Estado de México Mexico

**Keywords:** In situ, In vitro, Livestock, Nitrogen degradation, Sheep

## Abstract

**Background:**

The high dependence of intensive ruminant production on soybean meal and the environmental impact of this crop encourage the search for alternative protein-rich feeds. The use of insects seems promising, but the extent of their ruminal protein degradation is largely unknown. This parameter has major influence not only on N utilization efficiency but also on the environmental burden of ruminant farming. In addition, although assessing ruminal N degradation represents a key first step to examine the potential of new feeds, it is a challenging task due to the lack of a reference method. This study was conducted to investigate the potential of 4 insects (*Tenebrio molitor, Zophobas morio, Alphitobius diaperinus* and *Acheta domesticus*) as alternative protein sources for ruminants, using 3 methodologies: 1) a regression technique based on the in vitro relationship between gas production and ammonia-N concentration; 2) a conventional in vitro technique of batch cultures of ruminal microorganisms, based on filtering the incubation residue through sintered glass crucibles; and 3) the in situ nylon bag technique. The in vitro intestinal digestibility of the non-degraded protein in the rumen was also determined. Soybean meal was used as a reference feedstuff.

**Results:**

Comparison of evaluation methods (regression, in vitro and in situ) did not allow to reliably select a single value of ruminal N degradation for the studied substrates, but all techniques seem to establish a similar ranking, with good correlations between methods, particularly between regression and in situ results. Regardless of the methodology, nitrogen from the 4 insects (with contents ranging from 81 to 112 g/kg of dry matter) did not show high ruminal degradation (41–76%), this value being always lower than that of soybean meal. Furthermore, the in vitro intestinal digestibility of non-degraded N was relatively high in all feeds (≥ 64%).

**Conclusion:**

Overall, these results support the potential of the 4 studied insects as alternative feedstuffs for ruminants. Among them, *T. molitor* showed the lowest and greatest values of ruminal N degradation and intestinal digestibility, respectively, which would place it as probably the best option to replace dietary soybean meal and increase the sustainability of ruminant feeding.

## Background

Climate change has fostered research on mitigating the environmental impact of intensive ruminant farming, which has been pointed as a contributor to water eutrophication, acid deposition, and excessive nitrogen excretion [1, 2]. This environmental burden would largely be explained by the relatively low efficiency of utilization of protein-rich feeds by ruminants [3–5].

In addition, most intensive livestock production systems are highly dependent on soybean meal as the source of dietary protein. In Europe, this dependence is particularly relevant due to the ban on the use of meat meal and analogues in ruminant feeding, which encourages the search for additional protein-rich feeds [6–8].

Insects have been suggested as promising alternatives to plant proteins traditionally offered to animals [9–11]. The easy adaptation of insect production to different countries and their role in waste bioconversion and reduced land-use [9] makes them more environmentally friendly sources of dietary protein for livestock than soybean meal. However, to date there are very few scientific studies evaluating the use of insect products in ruminant diets [12–15]. Providing this information could help to push insects higher on policymaker agendas in Europe and worldwide and develop a regulatory framework for the authorization of insects as ruminant feed [9, 10].

Ruminal digestion largely determines the efficiency of utilization of dietary protein; knowing its degradation by rumen microbes is needed for estimation of the protein actually reaching the intestine and for prediction of protein requirements in ruminant production [5, 16, 17]. Thus, assessing the extent of protein degradation represents a first step to examine the potential of new feeds for ruminants, but available information on insect protein evaluation seems extremely scarce in the literature and it is mainly focused on silkworm (*Bombyx mori*) [18, 19].

Furthermore, protein evaluation of alternative feeds for ruminants is challenging: methods to estimate protein or nitrogen degradation based on in situ nylon bag incubations [16] and in vitro batch cultures of ruminal microorganisms [20] have often been questioned due to the likely overestimation caused by the loss of small particles through the pores (of either the bags or the crucibles employed in these techniques) without actually being degraded [21]. Another difficulty in measuring dietary nitrogen degradation arises from the simultaneous synthesis of ruminal microbial protein [5]. To avoid these problems, Raab et al. [17] proposed an alternative evaluation method based on linear regression between gas production and ammonia-N concentration in in vitro ruminal incubations. However, considering the few existing publications on this issue [22, 23], this proposal does not seem to have received much attention.

Thus, the value of 4 insect meals (from *Tenebrio molitor*, *Zophobas morio*, *Alphitobius diaperinus*, and *Acheta domesticus*) as protein sources for ruminant feeding was examined. To this aim, 3 different methodologies to estimate ruminal degradation were compared: 1) a regression technique based on the in vitro relationship between gas production and ammonia-N concentration; 2) a conventional in vitro technique of batch cultures of ruminal microorganisms, based on filtering the incubation residue through sintered glass crucibles; and 3) the in situ nylon bag technique.

## Methods

### Experimental animals

Four adult Merino ewes (body weight: 57.4 ± 8.2 kg), which were neither pregnant nor lactating, were equipped with a rumen cannula. Animals were offered a total mixed ration [dry matter (DM): 916 g/kg of fresh matter; crude protein (CP): 205 g/kg DM; neutral detergent fiber (NDF): 314 g/kg DM, consisting of dehydrated alfalfa, maize and barley grain, soybean meal, vitamins and minerals, with a forage:concentrate ratio of 60:40]. The ration was offered daily at 9:00 h, at approximately 1.1 times their maintenance energy requirements [24]. Barley straw and clean drinking water were always available.

### Experimental substrates

Four types of dehydrated insects were used: mealworms (*Tenebrio molitor*), morioworms (*Zophobas morio*), buffaloworms (*Alphitobius diaperinus*) and adult house crickets (*Acheta domesticus*). In addition, soybean meal was used as reference feedstuff. *Tenebrio molitor* was obtained from MealFood Europe (Doñinos de Salamanca, Spain) and the other three insects from Kreca Ento-Feed BV (Ermelo, the Netherlands).

The soybean meal and the insects were mixed with dry ice and then ground using an ultra-centrifugal mill (Retsch ZM 1000; Retsch Gmbh, Haan, Germany) through a 1-mm screen for in vitro incubations and through a 2-mm screen for in situ incubations. Dry ice was used to prevent the high fat content of feeds from affecting the process, embrittling the sample to achieve a homogeneous grind.

### Experimental procedures

#### Batch cultures of rumen microorganisms

Batch cultures of rumen microorganisms were performed as outlined previously [25] to carry out the protein evaluation using the regression technique described by Raab et al. [17], and a conventional in vitro method.

On the 3 incubation days, ewes were given free access to the diet for 3 h. Then, orts were removed and, 3 h later, samples of rumen digesta were collected through the cannula of each animal and filtered through 3 layers of cheesecloth. Drinking water had also been removed 1 h before rumen inoculum collection. Rumen fluids were immediately transferred to the laboratory in pre-warmed thermal flasks, where they were strained through a nylon membrane (250 μm of pore size; Fisher-Scientific S.L., Madrid, Spain) under a constant flow of CO_2_. Equal weights of the 4 strained rumen fluids were pooled and mixed (1:4) with phosphate-bicarbonate buffer solution [26]. The buffered rumen fluid (50 mL) was dosed in incubation flasks (125-mL), which contained 500 mg DM of substrate. Sealed flasks were incubated under anaerobic conditions for 16 h at 39.5 °C. Blanks containing buffered rumen fluid without substrate were also incubated under the same conditions. Incubations were repeated on 3 non-consecutive days (runs).

#### Regression technique

Following the in vitro conditions described above, each substrate was incubated in triplicate with 4 incremental levels of maize starch (Fluka 85,652, Madrid, Spain): 0, 100, 200 or 300 mg/flask. A total of 216 flasks were incubated: (5 incubation substrates + 1 blank) × 4 starch levels × 3 flasks × 3 incubation runs.

Head-space gas pressures were recorded at 4, 8 and 16 h of incubation. Accumulated gas pressure values were corrected for the amount of incubated DM and gas measured from blanks. Gas volumes were then estimated using a predictive linear regression equation derived from numerous simultaneous pressure and volume measurements [25].

After 16 h of incubation, the fermentation was stopped by placing the flasks into ice-water, and samples of buffered rumen fluid were centrifuged (at 976 × *g* for 10 min at 4 °C). The supernatant was acidified (1:1) with HCl 0.2 mol/L and stored at − 30 °C until ammonia-N analysis.

Relationships between gas production and ammonia-N concentration were used to estimate nitrogen degradation by linear regression (reg ND), as described by Raab et al. [17], with the modifications recommended by Mota et al. [22].

#### In vitro technique

Incubation residues from the flasks containing 0 mg of additional starch were filtered using pre-weighed sintered glass crucibles (100–160 μm; Pyrex, UK). A total of 54 flasks were filtered: (5 incubation substrates + 1 blank) × 3 flasks × 3 incubation runs.

Crucibles were dried in an air-forced oven (103 °C, 24 h) and residues were analyzed for nitrogen content to estimate N degradation (in vitro ND).

#### In situ technique

Ruminal nitrogen degradation was also estimated using the nylon bag technique [16]. To this aim, nylon bags (50 μm pore size; R1020, Ankom Technology Corp., Macedon, NY, USA) were filled with 6 g of each substrate and incubated for 16 h by suspending them in the rumen of each sheep (replicate) just before feeding. Five bags were incubated in each animal (1 bag/substrate) in a single day. After incubation, bags were removed, washed with cold water and stored frozen (− 20 °C, > 24 h) to facilitate the detachment of ruminal microorganisms from feed particles. Once defrosted, bags were washed again with cold water in an automatic washing machine (20 min) and dried in a forced-air oven (45 °C, 48 h). Nitrogen concentrations in the residues were analyzed to estimate N degradation (in situ ND).

The solubility of the DM and the N of each substrate was also estimated. After filling 2 bags per substrate with 6 g of feed, they were washed, dried and analyzed as described above for bags incubated in the rumen, with the exception that no in situ incubation was carried out.

#### In vitro intestinal digestibility

The in vitro intestinal digestibility of the non-degraded nitrogen (IDNDN) in the rumen was determined following the three-step in vitro procedure developed by Calsamiglia and Stern [27].

In situ incubation residues were used as the substrate for this test. From each sample, an amount equivalent to 15 mg of N was weighed into polypropylene tubes. Samples were first incubated (39.5 °C, 1 h) with 10 mL of an acid solution containing HCl (0.1 mol/L, pH: 1.9) and pepsin (1 g/L; P7012, Sigma-Aldrich, Madrid, Spain) to simulate abomasal digestion. After the pH was neutralized (using 0.5 mL of NaOH 1 mol/L), the second incubation (39.5 °C, 24 h) was conducted with 13.5 mL of a potassium phosphate buffer solution (0.3 mol/L, pH: 7.75) containing pancreatin (3 g/L; P7545, Sigma-Aldrich) to simulate intestinal digestion. Then, 3 mL of trichloroacetic acid was added to stop the digestion and precipitate non-degraded proteins. Samples were centrifuged (10,000 × *g*, 4 °C, 15 min) and the supernatant was stored at − 30 °C until analyzed for soluble nitrogen content.

### Chemical analyses

Samples of substrates were prepared (ISO 6498:2012) and analyzed for DM (ISO 6496:1999) and ash (ISO 5984:2002). The NDF and acid detergent fiber (ADF) contents were determined using an Ankom^2000^ fiber analyzer (Ankom Technology Corp. Methods 13 and 12, respectively). The NDF was assayed with sodium sulfite and alpha-amylase, and expressed with residual ash (the latter also for ADF). Ether extract (EE) was analyzed using the Ankom Filter Bag Technology and the technique described by AOCS [28].

Nitrogen concentration in substrates and incubation residues was analyzed (ISO 5983-2:2009) using a Kjeldahl autoanalyzer (Foss Kjeltec™ 2400, Hillerød, Denmark), whereas ammonia-N concentration in liquid incubation residues was determined by colorimetry [29].

### Statistical analyses

All statistical analyses were conducted with the SAS software package (version 9.4; SAS Institute Inc., Cary, NC, USA).

The reg ND was estimated by linear regression between gas production (*x*, mL) and ammonia-N (*y*, mg) using the REG procedure.

Other statistical analyses were performed using the MIXED procedure. Nitrogen degradation data were analyzed by ANOVA to test the fixed effects of evaluation method (i.e., reg ND, in vitro ND and in situ ND), of incubation substrate (i.e., soybean meal, *A. domesticus*, *A. diaperinus*, *T. molitor* and *Z. morio*), and their interaction. In vitro IDNDN and solubility data were analyzed by ANOVA to test the fixed effects of incubation substrate. Means were separated through the pairwise differences (“pdiff”) option of the least squares means (“lsmeans”) statement of the MIXED procedure, and adjusted for multiple comparisons using Bonferroni’s correction.

Pearson correlations between variables were examined using the CORR procedure.

Differences were declared significant at *P* < 0.05 and considered a trend toward significance at 0.05 ≤ *P* < 0.10. Least squares means are reported throughout the manuscript.

## Results and discussion

### Chemical composition of substrates

No statistical analysis was conducted to compare the chemical composition of substrates because each product derived from a single commercial batch.

As reported in Table 1, *A. domesticus* showed the greatest N concentration, which was close to that of *A. diaperinus* and 21% and 46% higher than those of *T. molitor* and *Z. morio*, respectively, whereas soybean meal showed similar values to *T. molitor*. Compared with other data of N concentration, obtained by reverse conversion from reported CP contents [30–32], our results were within the very wide ranges (in g/kg DM) for larvae of *T. molitor* (≈ 32–110), *Z. morio* (≈ 32–83), and *A. diaperinus* (≈ 96–130), and adults of *A. domesticus* (≈ 16–118). This high variability in N concentration may derive from potential differences in development stage, rearing process (in particular, diet composition) and N determination method [30–32]. We decided to compare nitrogen instead of protein concentrations because it would prevent possible bias due to the overestimation of CP contents obtained by using the conventional nitrogen-to-protein conversion factor of 6.25, which does not seem to apply to insect species [33–35].

**Table 1:** Chemical composition of experimental substrates, expressed in g/kg DM (except for DM itself; g/kg of fresh matter)^a^

	DM	OM	N	NDF	ADF	EE
Soybean meal	875	931	81	145	93	35
*Tenebrio molitor*	932	966	81	195	76	344
*Zophobas morio*	937	966	60	96	53	488
*Alphitobius diaperinus*	933	960	103	114	73	247
*Acheta domesticus*	913	947	112	134	84	181

^a^*DM* dry matter; *OM* organic matter; *N* nitrogen; *NDF* neutral detergent fiber; *ADF* acid detergent fiber; *EE* ether extract

Regarding other components, ether extract of *A. diaperinus* and *Z. morio* slightly exceeded previous reports [30, 31, 36], the latter species showing 2.7- and 14-fold greater fat content than *A. domesticus* and soybean meal, respectively. The moderate EE content of *A. domesticus* and the intermediate values of *T. molitor* seem consistent with mean values found in the literature [30, 31, 36].

As expected [30, 32, 37], fiber concentrations were relatively low in all substrates.

### Digestive utilization of nitrogen

Nitrogen, instead of protein, degradation was used as the estimation parameter in the 3 techniques of protein evaluation to avoid, as mentioned above, a possible bias due to the as-yet unclear nitrogen-to-protein conversion factors of the 4 insect products [33–35]. It is noteworthy that the analysis actually determines the content of N, which is then mostly transformed into protein by the conversion factor 6.25. However, in the case of insects, this factor overestimates the protein content [32] and studies based on amino acid analysis have proposed alternative conversion factors for larvae of different insect species, ranging from approximately 4.43 to 5.75 [33, 38, 39].

Table 2 reports the regression equations obtained using the methodology proposed by Raab et al. [17], with the modifications recommended by Mota et al. [22]. Intercepts indicate a relatively high potential nitrogen degradation when gas production would be zero (between 52% and 62% for soybean meal, *A. diaperinus* and *A. domesticus*, and 36–38% for *T. molitor* and *Z. morio*). Nevertheless, these results might be influenced by the composition of the ruminal inoculum, which was obtained from donor ewes consuming a diet very rich in CP (205 g/kg DM), and was collected 3–4 h after ingestion, which could have favored a high proteolytic activity during incubation [5, 40].

**Table 2:** Regression equations established between gas production (*x*, mL) and ammonia-N concentration (*y*, mg) after 16 h of in vitro incubation of substrates and increasing amounts of starch

	Regression equation	Adjusted R^2^	RMSE^a^
Soybean meal	*y* = 61.7–0.210 *x*	0.893	1.517
*Tenebrio molitor*	*y* = 35.7–0.136 *x*	0.927	1.105
*Zophobas morio*	*y* = 38.6–0.134 *x*	0.933	0.994
*Alphitobius diaperinus*	*y* = 51.7–0.092 *x*	0.768	1.747
*Acheta domesticus*	*y* = 55.7–0.133 *x*	0.905	1.449

^a^Root mean square error

**Fig. 1:**
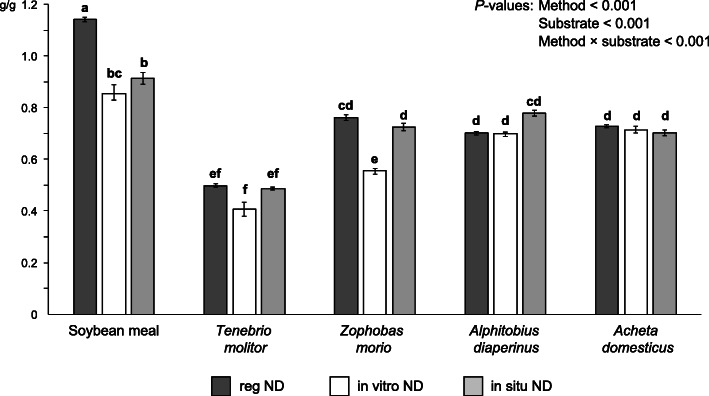
Nitrogen degradation (g/g) of the 5 incubation substrates using 3 different methods: regression (reg ND), in vitro (in vitro ND) and in situ (in situ ND). Vertical bars represent the standard error of the mean (SEM). Different letters (a-f) indicate significant differences (*P* < 0.05) for the interaction method × substrate

Estimated N degradation in the 5 incubation substrates, using the different methodologies, is shown in Fig. 1. Regardless the technique, lower values were found for the insects than for soybean meal (*P* < 0.001). Nitrogen degradation of this reference feed (> 85%) was higher than most available reports in the literature [17, 41, 42], which may be explained by the high proteolytic activity of ruminal microorganisms in our conditions, and the slightly longer incubation times (16 h in our case vs. 12 h in most available studies).

Among insects, the lowest N degradation was that of *T. molitor* (*P* < 0.001), a result that was consistently found with the 3 evaluation methods, with no differences between them (on average, 46%; *P* > 0.10). Neither were statistically significant differences due to methodology detected for *A. diaperinus* and *A. domesticus*, which also showed similar degradation values between methods (on average, 72%; *P* > 0.10). On the contrary, results for *Z. morio* were consistent when estimated by regression and in situ techniques (≈ 74%), but 25% lower in the in vitro estimation (56%; *P* < 0.001).

Major advantages of the in vitro technique are the high analytical capacity and low cost [5], but a greater variability in results is expected given the inherent characteristics of the method. In fact, it included an additional variation factor due to the need to scrape the crucibles to obtain the incubation residue on which N content is analyzed. Another concern is the potential underestimation of N degradation due to the increase of this compound with the growth of microorganisms (i.e., microbial protein synthesis) during incubations [43].

The regression method [17, 22] was proposed to avoid the confounding effect of microbial protein synthesis and, therefore, relatively higher N degradations would be expected. However, the illogical value of 114% degradation of soybean meal observed in our study (something that was not detected in any other case; *P* < 0.001) would help to explain why this technique has not spread widely.

In any event, a good agreement between regression and in situ N degradation results was obtained for the studied insects, with no differences when comparing values obtained with one or other methods (*P* > 0.10 in pairwise comparisons for each substrate). In addition, results from both techniques were highly correlated (*r* = 0.914; *P* = 0.030). The in situ nylon bag technique has been broadly used to examine ruminal degradation of protein-rich feedstuffs [16, 42, 44], but it is labor-intensive and difficult to standardize [5, 21]. In situ results also have the drawback related to the possible overestimation of N degradation due to the loss of soluble and small particles, a doubt that has also been raised for the in vitro technique and is far from recent [5, 22, 45]. In this regard, an important part of the degradation seemed to be due to solubility (see Table 3), which was always higher in insects than in soybean meal (for DM and N; *P* < 0.001). Among insects, the greatest N solubility was found for *Z. morio* and *A. domesticus,* and the lowest for *T. molitor* (*P* < 0.001). This solubility accounted for 25% of in situ ND of soybean meal, approximately 60% for *T. molitor* and *A. diaperinus*, and up to 74% for *Z. morio* and *A. domesticus*, with no significant correlation between both parameters (*r* = − 0.048; *P* = 0.939). In addition, although nitrogen solubility in the in situ method (Table 3) might have some analogy with the N degradation at zero gas production in the reg ND technique, neither was any correlation found between both results (*r* = − 0.173; *P* = 0.781).

**Table 3:** Solubility of dry matter and nitrogen of the 5 incubation substrates, g/g

	Incubation substrate						
	Soybean meal	*Tenebrio molitor*	*Zophobas morio*	*Alphitobius diaperinus*	*Acheta domesticus*	SED^a^	*P*-value
Solubility							
Dry matter	0.323^d^	0.487^c^	0.713^a^	0.477^c^	0.522^b^	0.006	< 0.001
Nitrogen	0.232^d^	0.289^c^	0.535^a^	0.471^b^	0.517^a^	0.006	< 0.001

^a-d^ Within a row, different superscripts indicate significant differences (*P* < 0.05) due to the effect of the incubation substrate

^a^*SED* standard error of the difference

The need to find a simple, fast, cheap and accurate method for protein evaluation of feeds for ruminants, specifically to determine their ruminal degradation, has been highlighted for a long time [5, 16, 22]. However, to date there is no agreement about the most convenient technique and, possibly, there is no single method of choice, which might depend on the specific characteristics of each feedstuff or study. In this regard, although our results would not allow to reliably select a single value of ruminal N degradation, the 3 methods do seem to establish a similar ranking of feeds and support a relatively low ruminal N degradation of the 4 insects. This is consistent with the few available data about *B. mori* [18, 46]. Correlation analysis also supported a significant relationship between in situ and in vitro ND (*r* = 0.926; *P* = 0.024) and a trend towards significance between in vitro and reg ND (*r* = 0.857; *P* = 0.064).

Because dietary protein escaping ruminal degradation is an effective way to increase duodenal protein flow [5], our results would support that replacing soybean meal by the studied insects, especially *T. molitor*, might be advantageous. Nevertheless, given that part of the non-degraded N in the rumen would probably derive from cuticular non-digestible nitrogen [32], results from the in vitro intestinal digestibility analysis seem crucial to support the potential of insects as feed for ruminants.

In this regard, the intestinal digestibility of non-degraded protein was high for all insects (Table 4), being greatest (*P* < 0.001) for *T. molitor* (78%), and lowest for *A. diaperinus* (64%), which did not show differences from soybean meal (*P* > 0.10). Using a methodology similar to that of our trial, Ioselevich et al. [18] found a lower digestibility for *B. mori* pupae (53%), whereas the in vivo study by Narang and Lal [47] reports an increase in apparent N digestibility when this latter insect replaced vegetable protein, which was associated with a trend towards greater body weight gain in calves. Compared with soybean meal, Jayanegara et al. [12, 13] observed a lower in vitro digestibility of DM for several insects, including *T. molitor*, but these authors employed the Tilley and Terry [48] technique and did not measure N degradation, which does not allow to discern if their results are explained by ruminal degradation (of N or other fractions) or subsequent digestion with pepsin. The apparent lack of other available data in the literature does not allow these inconsistent results to be attributed to insect species, methodologies or other factors.

**Table 4:** In vitro intestinal digestibility of the non-degraded nitrogen in the rumen (IDNDN, g/g) of the 5 incubation substrates

	Incubation substrate						
	Soybean meal	*Tenebrio molitor*	*Zophobas morio*	*Alphitobius diaperinus*	*Acheta domesticus*	SED^a^	*P*-value
IDNDN	0.680^bc^	0.782^a^	0.703^b^	0.640^c^	0.728^b^	0.018	< 0.001

^a-c^ Different superscripts indicate significant differences (*P* < 0.05) due to the effect of the incubation substrate

^a^*SED* Standard error of the difference

Overall, our results support the use of insects to replace soybean meal as a source of protein in ruminant feeding. Their lower ruminal N degradation might contribute to improve N utilization efficiency and, therefore, productivity while decreasing N excretion to the environment. Nevertheless, further protein evaluation studies are needed due to the high heterogeneity of these feeds and the limited available information. Determination of non-protein N in insects and the actual utilization of this fraction by ruminants would also be recommended.

## Conclusions

Comparison of evaluation methods (regression, in vitro and in situ) does not allow to reliably select a single value of ruminal N degradation for the studied substrates (soybean meal, *A. domesticus*, *A. diaperinus*, *T. molitor* and *Z. morio*), although all techniques seem to establish a similar ranking. Regardless of the methodology, nitrogen from the 4 insects (with contents ranging from 81 to 112 g/kg DM) would not show high ruminal degradation (41–76%), this value being always lower than that of soybean meal. Furthermore, the in vitro intestinal digestibility of non-degraded N in the rumen appears to be relatively high in all feeds (≥ 64%). Overall, these results support the potential of the 4 studied insects as alternative feedstuffs for ruminants. Among them, *T. molitor* showed the lowest and greatest values of ruminal N degradation and intestinal digestibility, respectively, which would place it as probably the best option to replace dietary soybean meal and increase the sustainability of ruminant feeding.

## Data Availability

The datasets from the current study are available from the corresponding author on reasonable request.

## Abbreviations

ADF: Acid detergent fiber
CP: Crude protein
DM: Dry matter
EE: Ether extract
IDNDN: Intestinal digestibility of the non-degraded nitrogen
NDF: Neutral detergent fiber
ND: Nitrogen degradation
OM: Organic matter
SED: Standard error of the difference
SEM: Standard error of the mean
